# Inventory of current practices regarding hematopoietic stem cell transplantation in metachromatic leukodystrophy in Europe and neighboring countries

**DOI:** 10.1186/s13023-024-03075-3

**Published:** 2024-02-07

**Authors:** Daphne H. Schoenmakers, Fanny Mochel, Laura A. Adang, Jaap-Jan Boelens, Valeria Calbi, Erik A. Eklund, Sabine W. Grønborg, Francesca Fumagalli, Samuel Groeschel, Caroline Lindemans, Caroline Sevin, Ludger Schöls, Dipak Ram, Ayelet Zerem, Holm Graessner, Nicole I. Wolf

**Affiliations:** 1 Department of Child Neurology, Amsterdam Leukodystrophy Center, Emma’s Children’s Hospital, Amsterdam UMC, Vrije Universiteit Amsterdam, Amsterdam, The Netherlands; 2 Cellular and Molecular Mechanisms, Amsterdam Neuroscience, Amsterdam, The Netherlands; 3 Medicine for Society, Platform at Amsterdam UMC, University of Amsterdam, Amsterdam, The Netherlands; 4 Hôpital La Pitié-Salpêtrière, Assistance-Publique Hôpitaux de Paris, Inserm U1127, Paris, France; 5 Division of Child Neurology, Children’s Hospital of Philadelphia, Philadelphia, USA; 6 Stem Cell Transplantation and Cellular Therapies Program, Department of Pediatrics, Memorial Sloan Kettering Cancer Center, New York, NY 10065 USA; 7 San Raffaele Telethon Institute for Gene Therapy (SR-TIGET), Pediatric Immunohematology Unit and Neurology and Neurophysiology Unit, IRCCS San Raffaele Scientific Institute, Via Olgettina, 60, 20132 Milan, Italy; 8 Pediatric Immunohematology Unit and BMT Program, IRCCS San Raffaele Scientific Institute, Via Olgettina, 60, 20132 Milan, Italy; 9 Section for Pediatric Neurology, Skåne University Hospital and Clinical Sciences, Lund, Lund University, 221 84 Lund, Sweden; 10 Center for Inherited Metabolic Diseases, Department of Pediatrics and Adolescent Medicine and Department of Clinical Genetics, Copenhagen University Hospital Rigshospitalet, Copenhagen, Denmark; 11 Department of Paediatric Neurology and Developmental Medicine, University Children’s Hospital, Tübingen, Germany; 12 Princess Máxima Center for Pediatric Oncology, Heidelberglaan 25, 3584 CS Utrecht, The Netherlands; 13 Wilhelmina Children’s Hospital, University Medical Center Utrecht, Lundlaan 6, 3584 EA Utrecht, The Netherlands; 14 Reference Center for Leukodystrophies, Pediatric Neurology Department, Hôpital Bicêtre, Le Kremlin Bicêtre, France; 15 Department of Neurology and Hertie Institute for Clinical Brain Research, University of Tübingen, Tübingen, Germany; 16 German Center of Neurodeenerative Diseases (DZNE), Tübingen, Germany; 17 Department of Paediatric Neurology, Royal Manchester Children’s Hospital, Manchester, UK; 18 Pediatric Neurology Institute, Dana-Dwek Children’s Hospital, Tel Aviv Sourasky Medical Center, Sackler Faculty of Medicine, Tel Aviv University, Tel Aviv, Israel; 19 Institute for Medical Genetics and Applied Genomics, Center for Rare Diseases, University of Tübingen, Tübingen, Germany

**Keywords:** Leukodystrophy, Metachromatic, Hematopoietic stem cell transplantation, Rare diseases, Europe, Healthcare disparities

## Abstract

**Background:**

For decades, early allogeneic stem cell transplantation (HSCT) has been used to slow neurological decline in metachromatic leukodystrophy (MLD). There is lack of consensus regarding who may benefit, and guidelines are lacking. Clinical practice relies on limited literature and expert opinions. The European Reference Network for Rare Neurological Diseases (ERN-RND) and the MLD initiative facilitate expert panels for treatment advice, but some countries are underrepresented. This study explores organizational and clinical HSCT practices for MLD in Europe and neighboring countries to enhance optimization and harmonization of cross-border MLD care.

**Methods:**

A web-based EUSurvey was distributed through the ERN-RND and the European Society for Blood and Marrow Transplantation Inborn Errors Working Party. Personal invitations were sent to 89 physicians (43 countries) with neurological/metabolic/hematological expertise. The results were analyzed and visualized using Microsoft Excel and IBM SPSS statistics.

**Results:**

Of the 30 countries represented by 42 respondents, 23 countries offer HSCT for MLD. The treatment is usually available in 1–3 centers per country (18/23, 78%). Most countries have no or very few MLD patients transplanted during the past 1–5 years. The eligibility criteria regarding MLD subtype, motor function, IQ, and MRI largely differ across countries.

**Conclusion:**

HSCT for MLD is available in most European countries, but uncertainties exist in Eastern and South-Eastern Europe. Applied eligibility criteria and management vary and may not align with the latest scientific insights, indicating physicians’ struggle in providing evidence-based care. Interaction between local physicians and international experts is crucial for adequate treatment decision-making and cross-border care in the rapidly changing MLD field.

**Supplementary Information:**

The online version contains supplementary material available at 10.1186/s13023-024-03075-3.

## Introduction

Metachromatic leukodystrophy (MLD) is a rare lysosomal storage disorder with an estimated birth prevalence of 1.4–1.8 per 100.000 [1–3]. Deficient arylsulfatase A activity leads to sulfatide accumulation affecting myelin of the central and peripheral nervous system. This central and peripheral demyelination leads to neurological deterioration and early death [4]. MLD is comprised of a spectrum of phenotypes based on the age at onset: late-infantile (LI, onset < 2.5 years old), early-juvenile (EJ, onset 2–6 years old), late-juvenile (LJ, onset ≥ 6–16 years old), and adult (onset ≥ 16 years old) [5]. Almost all MLD cases are caused by biallelic variants in *ARSA*; cases caused by variants in *PSAP* are extremely rare [6, 7]. The MLD field is rapidly evolving due to new innovations, such as the recently authorized ex vivo gene therapy in the European Union for LI and EJ MLD [8] and development of emerging guidelines and newborn screening programs [9, 10]. Gene therapy is not yet universally accessible nor approved in late-onset MLD. This means that hematopoietic stem cell transplantation (HSCT) is still a relevant pillar in the treatment of MLD.

HSCT has been used as a potential treatment in MLD since the 1980s. HSCT, by providing arylsulfatase-A producing donor myeloid cells, may slow or halt disease progression when offered presymptomatically or very early in the disease course. Despite early enthusiasm for this approach, overall, the outcomes have been mixed [5, 11, 12], with a lack of consensus regarding eligibility criteria and long-term outcomes [13–15]. When the disease is too advanced and brain white matter is irreversibly damaged, HSCT is not beneficial and may even trigger fast deterioration [16]. Efficacy of HSCT also depends on age of onset. Patients with late-infantile form have not benefited from HSCT, likely because of the rapidly disease progression [5, 17, 18]. For early-juvenile MLD it is unclear whether HSCT favors the outcome. Late-juvenile and adult forms with an onset above the age of 6 and 16 years old respectively, on the other hand, may be more likely to benefit from HSCT when performed before symptom onset or early in the disease course [13, 19–22], although long-term outcome may be less positive than previously thought. For example, ongoing central grey matter degeneration years post-transplantation has been found [15] as well as progressive peripheral neuropathy [14].

Clinical guidelines for MLD, also providing eligibility criteria for HSCT, are in progress, but not yet available. Current clinical practice, therefore, largely relies on the limited available literature and expert recommendations. The European Reference Network for Rare Neurological Disorders (ERN-RND) and the MLD initiative (MLDi), which is an international MLD registry and collaborative network, currently facilitate international expert discussions in which consensus-based individual treatment advice can be provided to participating countries [23, 24]. That the question of eligibility is relevant, is illustrated by the fact that (without newborn screening), the majority of patients, about two thirds, is too advanced for HSCT at diagnosis [18].

Beyond large leukodystrophy centers, it is not known whether treatment is centralized and whether eligibility criteria are followed. Many countries, even within Europe, are underrepresented in MLD-related working groups within the MLDi and ERN-RND. This leads to unequal access to knowledge, care, and expertise, which is particularly challenging in a rapidly changing rare disease field like MLD.

In this study, we explore the current practices regarding HSCT in MLD, in terms of organization of care and eligibility criteria used in Europe. This survey intends to contribute to the optimization and harmonization of (cross-border) care for MLD patients.

## Methods

The MLDi [23] expert group and ERN-RND [24], consisting of neurologists, pediatric neurologists, metabolic physicians, and hematologists/transplant specialists initiated this exploratory inventory. A quantitative questionnaire-based method was chosen to easily distribute the questionnaire and cover a large geographical area, i.e., at least one center in all countries in Europe. The design of the questionnaire was based on items usually considered by the MLDi expert group in clinical decision-making around HSCT in MLD [23] and the previously published Delphi procedure [5]. Three questions assessed the demographic characteristics of the respondents. Six questions (1 open-ended and 5 closed questions) elicited information on the organizational aspects of the care around HSCT and three questions addressed transplant regimen practices. Another ten closed questions addressed the clinical decision-making on eligibility, including applied thresholds for gross motor function and intelligence. Also the use of the gross motor function classification for MLD (GMFC-MLD) [25], a commonly used clinical scoring system, as eligibility criterion was explored. Because not all centers are familiar with the GMFC-MLD, a separate question about the ability to walk was used. The severity of MRI abnormalities assessed with the MLD MRI severity score as eligibility criterion was also queried [26]. After that, the participants were asked to make a treatment decision for four short case descriptions. The questionnaire ended with contact information about the European eligibility discussion panels. The complete questionnaire can be viewed in Additional File 1 (Additional files).

Responses were collected using the web-based survey software EUSurvey (EU Survey Version v1.5.2.9, 30/11/2022 14:44). The questionnaire was distributed through the website and newsletter of European Reference Networks for Rare Neurological Disorders and the emailing list of the Inborn Errors of Metabolism Working Group from the European Society for Blood and Marrow Transplantation (EBMT). Besides that, personal invitations followed by 1-3 reminders were sent to 89 physicians from different countries with either expertise in (pediatric) neurology, inherited metabolic diseases or hematology. Those physicians were either part of the MLDi, members of the Committee of National Advisors in Paediatric Neurology from the European Paediatric Neurology Society, or were recommended as MLD expert/national referral center by the invited physicians. No personal invitations could be sent to physicians in 5 European countries (Malta, Kosovo, Montenegro, North-Macedonia, Belarus) because no contact person could be identified. Responses were collected between 23-1-2023 and 22-3-2023.

The results of the closed questions were quantitatively analyzed and visualized using Microsoft Excel and IBM SPSS statistics 28. The open-ended question was qualitatively analyzed by organizing and categorizing the responses. The questions on the organizational aspects of care and clinical decision-making were analyzed on the level of countries. Additional inquiries were made in case of conflicting answers from multiple respondents from one country. The questions on the technical transplant aspects were analyzed by transplant centers or transplant units in case of separate pediatric and adult units. The responses on the four case descriptions were analyzed on the level of the individual respondents.

To provide a concrete idea about the estimated numbers of MLD patients born per year per country, those numbers were calculated based on the total yearly livebirths and the estimated birth prevalence of MLD of 1:40.000 [1, 2, 27]. The total livebirths per year were extracted from Eurostat [28], except for Israel and Kazakhstan for which national public demographic databases were consulted [29, 30]. The most recently reported total livebirths were used, being 2021 for most countries and all between 2018 and 2021.

## Results

From the 43 personally invited country representatives, 30 countries responded: Albania, Armenia, Austria, Belgium, Czech Republic, Denmark, Estonia, Finland, France, Georgia, Germany, Greece, Ireland, Israel, Italy, Kazakhstan, Latvia, the Netherlands, Norway, Poland, Portugal, Serbia, Slovak Republic, Slovenia, Spain, Sweden, Switzerland, Turkey, Ukraine, and the United Kingdom. For some countries, multiple representatives responded resulting in a total of 42 respondents from 38 different centers. There were 13 countries (Bulgaria, Croatia, Cyprus, Hungary, Lithuania, Luxembourg, Romania, Bosnia and Herzegovina, Azerbaijan, Iceland, Moldavia, Russia, Kyrgyzstan) whose contacted representatives did not respond. All following results must be seen in the light of the rarity of MLD. To illustrate this, the expected number of MLD patients born per year for all responding countries are provided in Table 1.

**Table 1:** Estimated number of MLD patients born per year

Country	Estimated number of MLD patients born per year
Turkey	28
Germany	20
France	19
United Kingdom	18–19
Kazakhstan	11
Italy	10
Spain	8–9
Poland	8–9
Ukraine	7
Israel	4–5
Netherlands	4–5
Belgium	3
Sweden	3
Czech Republic	3
Switzerland	2
Austria	2
Greece	2
Portugal	2
Denmark	1–2
Serbia	1–2
Ireland	1–2
Slovak Republic	1–2
Norway	1–2
Finland	1
Georgia	1
Armenia	1
Albania	0–1
Slovenia	0–1
Latvia	0–1
Estonia	0–1

Estimated number of MLD patients born per year calculated based on the total yearly livebirths and the estimated MLD birth prevalence of 1:40.000

### Availability and reimbursement of HSCT for MLD and number of transplants

**Fig. 1:**
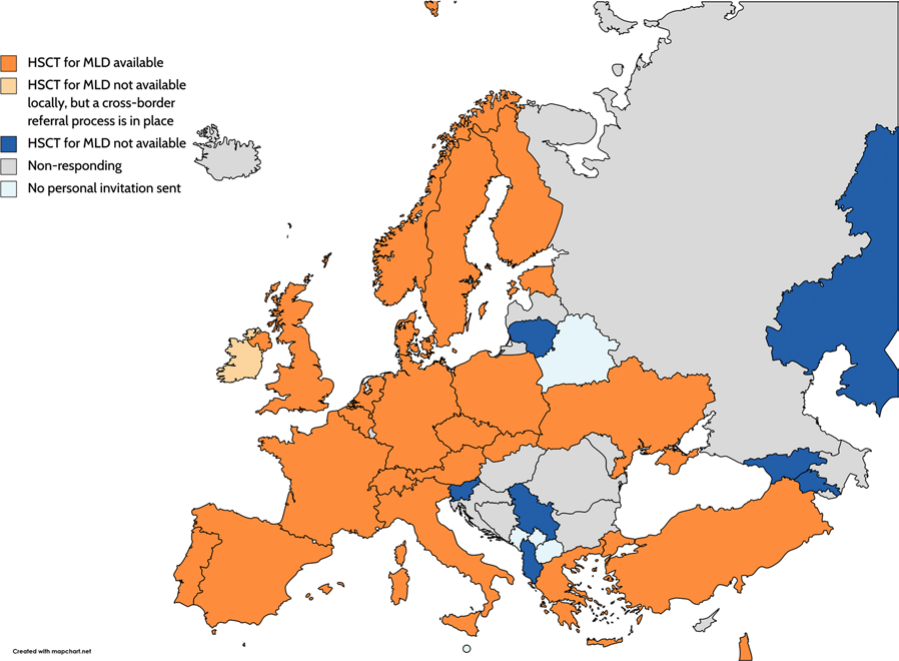
Geographical visualization of the availability of HSCT for MLD. Figure is created with mapchart.net

HSCT for MLD is performed in 23 (31 individual respondents) of the 30 responding countries (42 individual respondents). In total, 18 transplanting centers responded. HSCT is reimbursed in all countries except for Poland where the respondent stated that it is unknown, and for Serbia where there is no reimbursement for HSCT (Fig. 1). The number of transplants in total, for non-malignant diseases, for inborn errors of metabolism, and for MLD are shown in Table 2. The majority of the countries and centers have no or only very few MLD patients transplanted during the past year or five years, ranging from 0 to 4 patients during the past year and 0–8 during the past five years. Thirteen (72%) transplant centers indicated that they had not transplanted an MLD patient during the past year, and 8 (44%) transplant centers had not transplanted an MLD patient during the past five years.

**Table 2:** Estimated number of transplants per center

	Mean number of HSCT per center by indication Mean (SD; min–max) n = 18
Total	48.7 (43.4; 0.2–140)
Non-malignant diseases per year	11.4 (7.5; 0.5–28)
Inborn errors of metabolism per year	2.5 (2.3; 0.5–10)
MLD past year	0.5 (1.0; 0–4)
MLD past five years	1.5 (2.1; 0–8)

### Organization of care

In 18 of the 23 countries where HSCT for MLD is available, the HSCT care for MLD is centralized in one (n = 11), two (n = 3), or three (n = 4) centers (Table 3**)**. Five countries indicated that the transplant care for MLD is not centralized. Nine out of 18 of the responding transplant centers were accredited by the Joint Accreditation Committee ISCT-Europe & EBMT (JACIE) [31].

**Table 3:** Overview of current practices countries where HSCT for MLD is available

	N (%)
Total number of countries	23 (100%)
Centralization of HSCT care	
No	6 (22%)
1 center	11 (48%)
2 centers	3 (13%)
3 centers	4 (17%)
Referral by (pediatric) neurologist	21 91%)
Urgency of transplantation	
Never	3 (13%)
In all cases*	10(43%)
Only in near-symptomatic patients*	11(48%)
Routinely testing of siblings	
No	1 (4%)
All siblings	19 (83%)
Only younger siblings	1 (4%)
Other/no established routine	2 (9%)
Centralized clinical decision-making in one or two centers	9 (39%)
Eligible MLD subtypes	
Late-infantile	9(39%)
Early-juvenile	19(82%)
Late-juvenile	18(78%)
Adult	14(61%)
Aspects that are taken into account	
Severity of symptoms	22 (96%)
Donor availability	17 (74%)
Total IQ as criterion	
No	7 (30%)
> 60	1 (4%)
> 70	3 (13%)
> 75	1 (4%)
80	4 (17%)
85	6 (26%)
> 90	2 (9%)
GMFC-MLD as criterion	
No	9 (39%)
1	4 (17%)
< 2	10 (43%)
< 3	2 (9%)
MLD MRI severity score as criterion	
No	9 (39%)
< 7	5 (22%)
< 17	9 (39%)
Total number of units^	20 (100%)
Preferred source of CD34 + cells	
BM	8 (40%)
BM; UCB	3 (15%)
BM, UCB, PBSC	1 (5%)
BM; PBSC	2 (10%)
UCB	3 (15%)
PBSC	1 (5%)
Other	2 (10%)
Conditioning regimen	
Bu-Flu	9 (45%)
Bu-Cy	2 (10%)
Treosulfan-based	4 (20%
Reduced intensity	2 (10%)
Other	1 (5%)
Screening for carrier status in familial donors	15 (75%)

*One country has 1 center in which all cases are considered urgent and 1 center only in near-symptomatic patients

^In total 20 transplant units from 18 centers. From 2 centers both the adult and pediatric transplant unit responded, for some aspects different protocols were applied

Patients are most commonly referred to the transplant center by (child) neurologists (21/23, 91%). After referral, the transplantation was considered urgent (transplant within 4–8 weeks) in all cases in 10 centers, and urgent in (near-)symptomatic patients according to 11 centers. Three centers did not consider MLD an urgent indication for HSCT because it is a non-malignant disease.

Nineteen out of 23 countries routinely tested all siblings of index patients to identify presymptomatic patients. In one country only younger siblings are tested, and one other country did not test siblings. The remaining two countries did not answer this question.

### Transplant protocols and donor screening

The standard transplant regimen was comparable in most centers. Bone marrow was the preferred source of CD34 + positive cells in 8 transplant units. A busulfan-fludarabine regimen was the preferred conditioning regimen in 9 transplant units. In 13 transplant units, familial donors were screened for MLD carrier status to exclude heterozygous carriers from donation.

### Clinical decision-making

In 9/23 countries, clinical decision on whether or not to treat a MLD patient with HSCT is concentrated in one or two centers per country. Respondents from 20 countries indicated that a multi-disciplinary team involving both transplanters and neurologists decides whether an MLD patient is eligible for treatment.

The potential eligibility per MLD subtype differed across the countries, with late-infantile MLD being considered eligible in 9 countries, early-juvenile in 19 countries, late-juvenile in 18 countries, and adult in 14 countries (Table 3). Also practices with regard to symptom status differed across countries. Respondents from 9 countries indicated that pre- or early-symptomatic patients regardless of MLD subtype can be considered as eligible, whereas respondents from 12 countries considered only patients that are pre- or early-symptomatic and have juvenile or adult MLD as eligible. A single respondent from one country indicated that all patients with a confirmed diagnosis of MLD regardless of symptom status and MLD subtype are considered eligible for HSCT. In contrast, one respondent from another country indicated that only patients that are pre-symptomatic regardless of MLD subtype can be considered eligible.

Twenty-four respondents from 20 countries did not know whether patients with pathogenic variants in *PSAP* instead of *ARSA* would be considered eligible for HSCT. The remaining 6 respondents indicated that those patients would not be considered as eligible.

In deciding whether a patient will be treated with HSCT, other aspects were usually considered as severity of symptoms (30/31, 97% respondents; 22 countries) and donor availability (22/31, 71% respondents; 17 countries).

### Eligibility criteria

Intelligence quotient (IQ) was a key eligibility criterion according to 23 respondents (16 countries). The most common standardized IQ score thresholds were > 85 (6 countries) and > 80 (4 countries). Other thresholds included: > 60 in 1 country, > 70 in 3 countries, > 75 in 1 country, and > 90 in 2 countries. Three respondents that selected the > 85 cut-off for total IQ commented that decisions are made case-by-case and that a lower IQ may be accepted in selected cases.

**Fig. 2:**
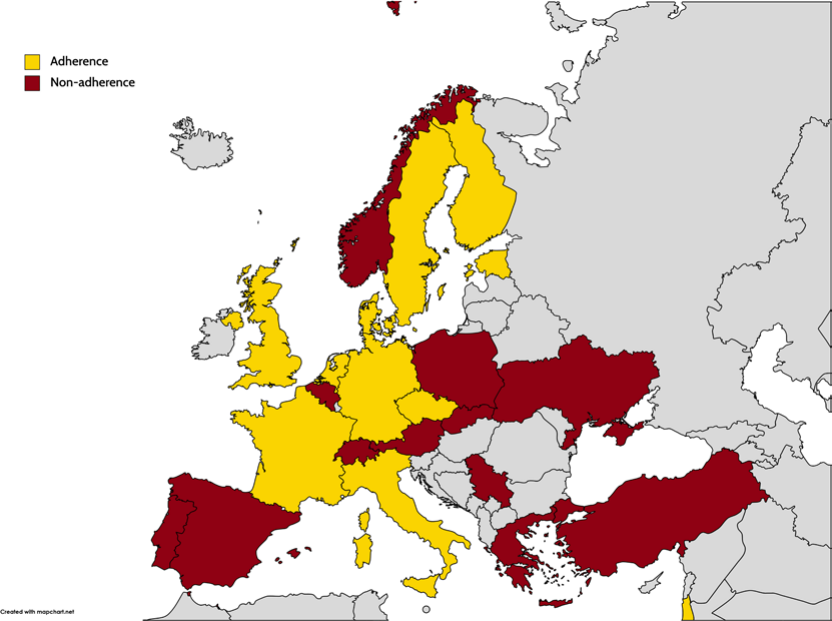
Geographical visualization of adherence or non-adherence to the usually adopted eligibility criteria. Adherence was defined as (1) juvenile and adult patients are considered eligible, (2) IQ > 70 and (3) GMFC-MLD ≤ 2 or able to walk with support. Non-adherence was defined as (1) late-infantile patients considered eligible, (2) IQ below 70 eligible or (3) GMFC-MLD > 2 or not able to walk eligible. Figure is created with mapchart.net

Gross motor function was also a common criterion for HSCT eligibility. Of the 20 respondents from 16 countries that utilize GMFC-MLD as an eligibility criterion, an eligibility threshold of a score < 1 was used in 4 countries, a score < 2 in 10 countries, and a score < 3 in 2 countries. Six countries reported that no restrictions for the ability to walk were used or that this was not part of the eligibility criteria. The ability to walk without support was a criterion in 15 countries. The ability to walk with light support of two hands or a walking aid was a criterion in 3 countries and 1 country, respectively. A visual representation of the differences across countries in terms of eligible MLD subtypes, motor function and IQ is provided in Fig. 2. The severity of abnormalities on brain MRI was not part of the eligibility criteria in 9 countries. Respondents from 5 countries responded that patients were only eligible when the MLD MRI severity score is < 7 and 9 countries use a cut-off < 17.

**Fig. 3:**
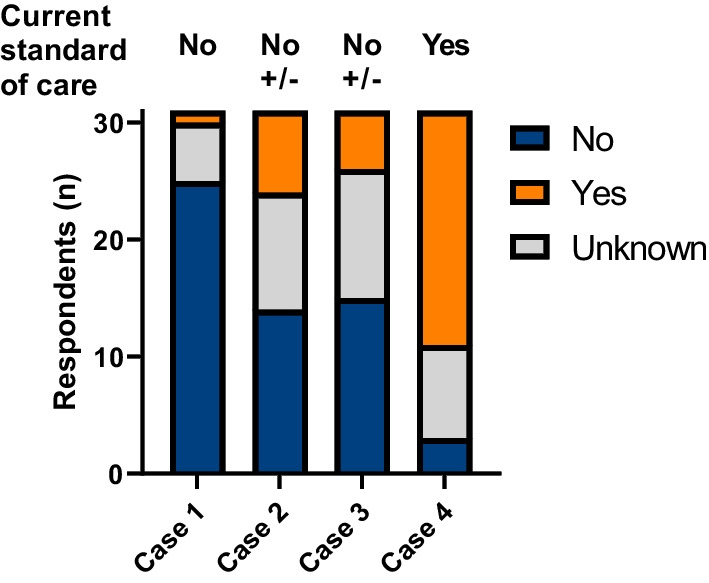
Responses on current clinical practice for sample cases. The stacked bars show the numbers of respondents that would likely treat with HSCT (yes) or not likely treat with HSCT (no) according to the current clinical practice in their country. For each case the current standard of care according to the expert centers is shown above the bars, in which ‘ ± ’ indicates a great uncertainty regarding standard of care

Finally, each respondent was provided 4 representative clinical cases (Table 4) to capture regional differences in management. The sample cases emphasize the heterogeneity of treatment advice for the representative cases. Only in case 1 and 4 a clear majority voted for not to treat / to treat respectively (Fig. 3).

**Table 4:** Sample cases

Case 1
• 2-year-old child • Presents with losing motor milestones, not able to sit without support anymore, Never learned to walk independently • Able to speak single words • GMFC-MLD 4, no IQ score available, MLD-Loes 13
Case 2 • 9-year-old child • Presents with decline in school performance during past 3 years and recently frequent falls • Normal initial development, goes to regular school • GMFC-MLD 1, TIQ 72, MLD-Loes 18
Case 3 • 17-year-old adolescent • Presents with decline in school performance (started at regular pre-university school and currently special education) and behavioral disturbances, increasingly present during past 5 years • No motor problems • GMFC-MLD 0, TIQ 63, MLD-Loes 23
Case 4 • 22-year-old patient • No symptoms • Diagnosed through a family screening because of an affected sibling (28 y/o) • GMFC-MLD 0, TIQ 106, MLD-Loes 16

## Discussion

HSCT is available and well-established for MLD patients in the majority of western European countries. Despite its widespread availability, clinical practices in terms of clinical decision-making and applied eligibility criteria differ across countries. The transplant regimen, on the other hand, is relatively harmonized, which is most likely explained by the widely adopted international standards for hematopoietic cell treatment [32].

All responding countries and centers transplanted none or only few patients during the past year or past five years, even the largest centers. There was considerable heterogeneity regarding eligibility and donor selection. We hypothesize that may be because of the rarity of MLD, limitations on eligibility with symptomatic disease, and a lack of consensus guidelines. Some respondents explicitly stated that they have insufficient experience to be able to properly complete the questionnaire. It should also be noted that some respondents might have a clinical experience restricted to either children or adults, which are significantly different presentations in MLD. Overall, there seems to be a prominent sense of uncertainty on when to offer HSCT to MLD patients, emphasizing the need for clinical guidelines or a care pathway for MLD [33]. This is especially true for straight-forward cases as discussed in sample cases 1 and 4 where, according to current common practice, HSCT is not advised for case 1 because it is a clearly symptomatic late-infantile patient, but is indicated for case 4 as a pre-symptomatic adult patient [5, 17, 22]. Despite this, 20% of the respondents in case 1 and 38% in case 4 were not sure how to proceed or selected the opposite of standard management.

This survey also underscored clinical uncertainties regarding the overall effectiveness of HSCT and thresholds for HSCT eligibility. Whereas treatment in the pre-symptomatic stage is reported in several cohorts of late-onset patients to have good outcome [13, 18, 22], treatment with first symptoms remains inconclusive regarding inclusion criteria, as demonstrated in borderline sample cases 2 and 3. In younger children, the gene therapy development program recommended an IQ ≥ 85 as eligibility criterion [8], which was the most common threshold reported in the centers for HSCT [16]. A total IQ below 85 in the clinical vignette of case 2, together with a suspected peripheral neuropathy causing frequent falls, would advocate against beneficial effects of HSCT. For older patients, there is evidence that some patients have an insidious onset with extremely slow cognitive decline and spared motor function, as illustrated in clinical vignette of case 3 [34–36]. The effects of HSCT in this (already symptomatic) late-onset patient group are insufficiently researched, and even less is known about clear-cut HSCT eligibility criteria.

The European network of MLD expertise, e.g., the MLD initiative and the ERN-RND guideline working group for MLD, typically involves a limited number of Western, Southern and Northern European countries. Key MLD experts from these countries (Denmark, Germany, Israel, Italy, the Netherlands, Spain, Sweden, the United Kingdom) participated in this inventory. Similar to the previously published distribution of ERN full members, these countries also have the highest number of ERN full members overall and relative to population size [37]. Gaining insight into the clinical practices regarding HSCT in MLD in underrepresented countries in Europe was challenging and may have impacted the reliability of this inventory. For some countries, it was difficult or impossible to reach a physician involved in care for MLD patients despite several avenues of approach. We hypothesize that this could also reflect the challenges of families to find a physician with the necessary expertise. Still, with the availability of gene therapy, a highly specialized treatment that is administered in a limited number of European countries, and emerging newborn screening programs, streamlining cross-border care and offering a standard level of expertise dealing with MLD to families is gaining even more urgency.

In conclusion, physicians in Europe adopt different clinical practices regarding HSCT for MLD. This may indicate that physicians struggle with providing and arranging evidence-based care for patients with the rare disease MLD, for which treatments emerge, but clinical guidelines are still under development. An imminent risk is significant cross-country differences in clinical practices regarding HSCT in MLD with, as a result, unequal access to high-quality care and healthcare disparities.

The results of this survey lead to some easily implemented suggestions improving the care for MLD patients. First, all siblings of an index patient with MLD, regardless of age, should be tested to identify possible asymptomatic patients. Second, potential family donors should be screened for carrier status as arylsulfatase A activity is lower in heterozygous carriers [11]. Third, international interaction between physicians and MLD experts, e.g. by expert panels, is crucial for appropriate treatment decision-making and provides expertise regardless of living place. Fourth, cross-border care for these highly specialized treatments would benefit from embedding in national and international health policies.

### Supplementary Information

**Additional file 1**. Questionnaire and sample cases.

## Data Availability

The dataset used in current study is available in anonymized form upon reasonable request. The dataset cannot be shared openly because of privacy reasons. We did not ask permission for sharing the dataset openly and we are unable to contact all respondents again because we have not collected personal contact information.
