# Supplementary material for: Inventory of current practices regarding hematopoietic stem cell transplantation in metachromatic leukodystrophy in Europe and neighboring countries
Source: Orphanet J Rare Dis. 2024 Feb 7;19:46. doi: 10.1186/s13023-024-03075-3 (PMC10848395; doi:10.1186/s13023-024-03075-3)
Supplement: Supplementary file 1 — Additional file 1. Questionnaire and sample cases. [file 13023_2024_3075_MOESM1_ESM.docx]

# Additional file 1 - Questionnaire

INTRODUCTION

In this questionnaire, access to HSCT, organizational aspects and clinical decision-making on whether or not to treat with HSCT in MLD are investigated. Please use your experience to fill out the questionnaire. There is no wrong or right in this questionnaire and the responses will only be used for this exploratory research.

BASIS INFORMATION

1. What is your expertise/function?
   1. Bone marrow transplant specialist
   2. Specialist inherited metabolic diseases
   3. (pediatric) neurologist
   4. Other, namely…
2. In which country is your center of employment located?
3. What is the name of your center of employment?
   1. *Please note that this information will only be used to monitor the responses and that this information will not be published.*

HSCT practices

1. Is HSCT for MLD available/being done in your country?
   1. Yes
   2. No 🡪 If bone marrow transplantations for MLD are never done in your country you do not need to answer the next questions of this survey.
2. To which center or country do you refer MLD patients for potential treatment with HSCT?
3. Are HSCTs in MLD patients done in your center?
   1. Yes
   2. No
4. How many HSCT per year are done in your center?
5. How many HSCT for non-malignant diseases are done per year in your center?
6. How many HSCT for inborn errors of metabolism are done per year in your center?
7. How many HSCT for MLD are done in your center during:
   1. The past year:
   2. The past 5 years:
8. Optional: elaborate on applicable quality criteria in your country.
9. What is the preferred source of CD34+ in IEM/neurometabolic diseases at your center:
   1. Bone marrow (BM)
   2. Cord blood (CB/UCB)
   3. Peripheral blood stem cells (PBSC)
   4. Other, specify…
10. What type of conditioning is the preferred choice for MLD at your center?
    1. Bu/FLU
    2. Bu/cy
    3. Reduced intensity
    4. Treosulfan based regimen
    5. Other, specify...
11. Do you screen familiar donors for carrier status to exclude them from donation?
    1. Yes
    2. No

ORGANISATION OF HSCT CARE

1. Is HSCT for MLD reimbursed in your country?
   1. Yes
   2. No
   3. Unknown
2. Is the HSCT care for MLD patients in your country centralized in one or a few dedicated centers?
   1. Yes
   2. No
3. How many centers are transplanting MLD patients in your country?
4. Optional: Who is the contact person for the above mentioned transplant center? In case multiple centers transplant MLD patients, please provide multiple contact persons.
5. Are there separate centers/transplant units for transplanting children and adults?
   1. Yes
   2. No
6. How does the patient usually arrive at the HSCT center?
   1. The family contacts the center of choice directly
   2. The neurologists/neuropediatricians refers patient to the HSCT center
   3. Through standardized referral process. > describe briefly
   4. Other, namely…

CLINICAL DECISION-MAKING

1. How many centers in your country do the clinical decision-making on whether or not to treat an MLD patient with HSCT?
2. How is decided whether a patient is eligible for HSCT in your country?
3. After the decision has been made, is transplant for MLD considered/managed as urgent at your center (if possible performed within 4-8 weeks including work-up)
   1. Yes, always
   2. Yes, in case of symptomatic patients or patients close to their expected disease onset
   3. Never (e.g. because it is not a malignant disease)
   4. Optional: elaborate on timelines in exceptional circumstances like NBS identified patients
4. Who decides if a patient is eligible for HSCT in your country?
   1. Patient/parents/family
   2. Referring physician
   3. Physician with special expertise in MLD
   4. Transplant specialist/hematologist
   5. Multi-disciplinary discussion with HSCT- and neurological team
   6. Other, specify…
5. Which MLD subtypes are eligible for HSCT in your country?
   1. Late-infantile (onset <2,5 years old)
   2. Early-juvenile (onset 2,5 - 6 years old)
   3. Late-juvenile (onset 6-16 years old)
   4. Adult (onset >16 years old)
6. Which MLD patients are considered to be eligible for HSCT in daily clinical practice in your country?
   1. All patients with a confirmed diagnosis of MLD regardless of symptom status and MLD subtype
   2. Only patients that are pre- or early symptomatic (>specify in next question) regardless of MLD subtype
   3. Only patients that are pre- or early symptomatic (>specify in next question) and have juvenile or adult MLD.
   4. Only patients that are pre-symptomatic regardless of MLD subtype
   5. Only patients that are pre-symptomatic and have juvenile or adult MLD.
7. Are MLD patients with pathogenic variants in *PSAP* instead of *ARSA* considered to be eligible for HSCT in daily clinical practice in your country?
   1. Yes
   2. No
   3. Unknown
8. Which eligibility criteria are used for the following clinical features? Please focus on disease progression and assume that abilities are age appropriate. MLD patients are eligible for HSCT if…
   1. Total IQ is
      1. No restrictions/not part of the criteria used in my center
      2. > 40
      3. > 50
      4. > 60
      5. > 70
      6. > 75
      7. > 80
      8. > 85
      9. > 90
   2. If you use different IQ cut-offs for different age groups, please elaborate.
   3. Gross motor function classification for MLD (GMFC-MLD)
      1. No restrictions/not part of the criteria used in my center
      2. <1
      3. <2
      4. <3
      5. <4
      6. <5
      7. <6
   4. Ability to walk
      1. No restrictions/not part of the criteria used in my center
      2. Can walk without support
      3. Can walk with light support of 2 hands
      4. Can walk with support
   5. Abnormalities on brain MRI quantified using MLD-Loes score
      1. No restrictions/not part of criteria in my center
      2. <7
      3. <17
      4. Other, namely..
   6. Other criteria namely…
      1. Specify…
9. Which aspects play a role in deciding whether a patient will be treated with HSCT? (checkbox/multi-select question)
   1. Patient’s/parents wish
   2. Donor availability
   3. Severity of symptoms
   4. Other, namely…
10. Do you routinely test siblings of an index patient?
    1. Only younger siblings
    2. All siblings
    3. No, we do not test siblings
    4. Other, namely…

SAMPLE CASES

1. Please decide for the following MLD patients if, according to the current clinical practice in your center/country, the patient would likely be treated with HSCT. (Please focus on clinical characteristics, we assume that the patient/family has no objection to transplantation and that a matching donor can be found)
   1. For all cases:
      1. Yes, this patient would likely be treated with HSCT
      2. No, this patient would not likely be treated with HSCT
      3. Unknown
   2. Case 1
      1. 2 year old child
      2. Presents with losing motor milestones, not able to sit without support anymore
      3. Never learned to walk independently
      4. Able to speak single words
      5. GMFC-MLD 4, no IQ score available, MLD-Loes 13
   3. Case 2
      1. 9 year old child
      2. Presents with decline in school performance during past 3 years and recently frequent falls
      3. Normal initial development, goes to regular school
      4. GMFC-MLD 1, TIQ 72, MLD-Loes 18
   4. Case 3
      1. 17 year old adolescent
      2. Presents with decline in school performance (started at regular pre-university school and currently special education)
      3. and behavioral disturbances, increasingly present during past 5 years
      4. No motor problems
      5. GMFC-MLD 0, TIQ 63, MLD-Loes 23
   5. Case 4
      1. 22 year old patient
      2. No symptoms.
      3. Diagnosed through a family screening because of an affected sibling (28 y/o).
      4. GMFC-MLD 0, TIQ 106, MLD-Loes 16
2. Extra question: Do you know that there is a European expert panel* that can be consulted to discuss the eligibility for GT and HSCT of MLD patients?
   1. Yes
   2. No

*This panel is an ad hoc experts’ meeting in which a patient’s eligibility for GT / HSCT will be plenary discussed with MLD experts. The referring physician presents the case, followed by a discussion and advice. If you face a MLD patient in your clinic that you would like to discuss regarding eligibility for GT and HSCT, please contact us: [MLDinitiative@amsterdamumc.nl](mailto:MLDinitiative@amsterdamumc.nl)

# Additional file 2 - Respondents

|  | (pediatric) neurologist | Specialist inherited metabolic diseases | Bone marrow transplant specialist | Internal medicine | Total  N=42 |
| --- | --- | --- | --- | --- | --- |
| Albania | 1 |  |  |  | 1 |
| Armenia | 1 |  |  |  | 1 |
| Austria | 1 |  |  |  | 1 |
| Belgium |  |  | 1 |  | 1 |
| Czech Republic |  | 1 | 1 |  | 2 |
| Denmark |  | 1 | 1 |  | 2 |
| Estonia |  | 1 |  |  | 1 |
| Finland | 1 |  |  |  | 1 |
| France | 1 | 1 | 1 |  | 3 |
| Georgia | 1 |  |  |  | 1 |
| Germany | 3 |  |  |  | 3 |
| Greece | 1 |  |  |  | 1 |
| Ireland |  | 1 |  |  | 1 |
| Israel | 1 |  |  |  | 1 |
| Italy |  | 1 | 1 |  | 2 |
| Kazakhstan | 1 |  |  |  | 1 |
| Latvia |  | 1 |  |  | 1 |
| Netherlands | 1 |  | 2 |  | 3 |
| Norway | 1 |  |  |  | 1 |
| Poland |  |  |  | 1 | 1 |
| Portugal | 1 |  |  |  | 1 |
| Serbia | 1 | 1 |  |  | 2 |
| Slovak Republic |  | 1 |  |  | 1 |
| Slovenia | 1 |  |  |  | 1 |
| Spain | 1 |  |  |  | 1 |
| Sweden |  |  | 1 |  | 1 |
| Switzerland | 1 |  | 1 |  | 2 |
| Turkey | 1 |  |  |  | 1 |
| Ukrain | 1 |  |  |  | 1 |
| United Kingdom | 2 |  |  |  | 2 |
